# Randomized placebo-controlled trial of feline-origin *Enterococcus hirae* probiotic effects on preventative health and fecal microbiota composition of fostered shelter kittens

**DOI:** 10.3389/fvets.2022.923792

**Published:** 2022-11-17

**Authors:** Jody L. Gookin, Sandra J. Strong, José M. Bruno-Bárcena, Stephen H. Stauffer, Shelby Williams, Erica Wassack, M. Andrea Azcarate-Peril, Marko Estrada, Alexis Seguin, Joerg Balzer, Gigi Davidson

**Affiliations:** ^1^Department of Clinical Sciences, College of Veterinary Medicine and Comparative Medicine Institute, North Carolina State University, Raleigh, NC, United States; ^2^Department of Environmental Services, Wake County Animal Center, Raleigh, NC, United States; ^3^Orange County Animal Services, Chapel Hill, NC, United States; ^4^Department of Plant and Microbial Biology, North Carolina State University, Raleigh, NC, United States; ^5^Veterinary Hospital Pharmacy, College of Veterinary Medicine and Comparative Medicine Institute, North Carolina State University, Raleigh, NC, United States; ^6^University of Wisconsin Veterinary Care, Madison, WI, United States; ^7^Department of Clinical Sciences, College of Veterinary Medicine, Mississippi State University, Starkville, MS, United States; ^8^Division of Gastroenterology and Hepatology, and UNC Microbiome Core, Department of Medicine, Center for Gastrointestinal Biology and Disease, School of Medicine, University of North Carolina, Chapel Hill, NC, United States; ^9^IDEXX Laboratories, Inc., West Sacramento, CA, United States; ^10^Vet Med Labor GmbH Division, IDEXX Laboratories, Inc., Kornwestheim, Germany

**Keywords:** 16S rRNA gene amplicon sequencing, diarrheal pathogens, growth and survival, infection, polymerase chain reaction, shelter medicine

## Abstract

**Introduction:**

Diarrhea is the second most common cause of mortality in shelter kittens. Studies examining prevention strategies in this population are lacking. Probiotics are of particular interest but studies in cats are largely limited to healthy adults or those with induced disease. Only one study in domestic cats describes the use of host-derived bacteria as a probiotic. We previously identified *Enterococcus hirae* as a dominant species colonizing the small intestinal mucosa in healthy shelter kittens. Oral administration of a probiotic formulation of kitten-origin *E. hirae* (strain 1002-2) mitigated the increase in intestinal permeability and fecal water loss resulting from experimental enteropathogenic *E. coli* infection in purpose-bred kittens. Based on these findings, we hypothesized that administration of kitten-origin *E. hirae* to weaned fostered shelter kittens could provide a measurable preventative health benefit.

**Methods:**

We conducted a randomized, placebo-controlled, blinded clinical trial to determine the impact of a freeze-dried *E. hirae* probiotic on body weight gain, incidence of diarrhea, carriage of potential diarrheal pathogens, and composition of the intestinal microbiota in weaned fostered shelter kittens.

**Results:**

One-hundred thirty kittens completed the study. Fifty-eight kittens received the probiotic and 72 received the placebo. There were no significant differences in age, weight upon initiation of the study, number of days in the study, average daily gain in body weight, or weight at completion of the study. Kittens treated with *E. hirae* were 3.4 times less likely to develop diarrhea compared to kittens treated with placebo (odds ratio = 0.294, 95% CI 0.109–0.792, *p* = 0.022). A significant impact of *E. hirae* was not observed on the presence or abundance of 30 different bacterial, viral, protozoal, fungal, algal, and parasitic agents in feces examined by qPCR. With exception to a decrease in *Megamonas*, administration of the *E. hirae* probiotic did not alter the predominant bacterial phyla present in feces based on 16S rRNA gene amplicon sequencing.

**Discussion:**

Decreased incidence of diarrhea associated with preventative administration of *E. hirae* to foster kittens supports a rationale for use of *E. hirae* for disease prevention in this young population at high risk for intestinal disease though additional studies are warranted.

## Introduction

It is estimated that 180 million kittens are born each year in the United States and hundreds of thousands of orphaned and abandoned kittens are fostered by U.S. animal shelters (1–3). Diarrheal disease is the second most common illness to afflict shelter kittens and many will die from diarrhea before they reach an age of 8 weeks (4, 5). The causes of diarrhea are likely multifactorial including an immature immune system, weaning stress, independent eating, change in food type, waning maternal antibodies, and increased exposure to diarrheal pathogens (1, 4, 6, 7). Efforts aimed at diagnosis and treatment of infectious causes of diarrhea in this population is challenging due to the large number and diversity of potential pathogens (viral, bacterial, parasitic and protozoal), frequent carriage of these pathogens by kittens without diarrhea, many agents for which there is no specific treatment, and limited resources. Consequently, orphan kittens that develop diarrhea are often treated empirically for intestinal bacterial, protozoal, or parasitic infections and are commonly provided supportive care in the form of antibiotics, probiotics, vitamin supplements, subcutaneous fluids, or assisted feeding (8).

Among the many knowledge gaps including causes and treatment outcomes of foster kitten diarrhea is a lack of studies examining prevention strategies for diarrhea in this population. In particular, probiotics (9) are of particular interest due to the multiple proposed beneficial effects such as promoting resistance to colonization by pathogens, stabilization of the intestinal microbiota, immunomodulatory effects on gut function, and metabolic impact on nutrient digestion. To date however, research examining the impact of probiotics in cats are mostly limited to investigations of healthy, adult-aged, purpose-bred cats, or cats with experimentally-induced disease (e.g., FIV and FHV-1) or administered antibiotics (10–20). Only 2 studies have examined the effect of probiotics on cats with diarrhea. In a study of older stray and feral cats housed short-term in a shelter setting, administration of a probiotic containing *Enterococcus faecium* SF68 did not protect against diarrhea but decreased the number of cats having diarrhea of ≥2 days duration (21). In a second study of adult cats with naturally-occurring chronic diarrhea, treatment with a synbiotic containing *Bifidobacterium, Enterococcus*, and *Lactobacillus* was reported to significantly improve fecal score (22).

In particular, the enterococci are components of the normal microbiota of the gastrointestinal tract and are ubiquitous in ecosystems ranging from soil, water, plants and food products (23). We have previously identified that a specific species of enterococcus, *Enterococcus hirae*, is the principal species inhabiting the mucosa-associated microbiota of the small intestine in healthy fostered shelter kittens (4) and is frequently observed to extensively colonize the surface of the intestinal epithelium (24). In kittens succumbing to severe illness, *E. hirae* are displaced by opportunistic and virulent members of enterococci such as *Enterococcus faecalis* and such kittens are more likely to be diagnosed with enteroadherent enteropathogenic *Escherichia coli* infection (EPEC) (4). Using a strain of *E. hirae*, cultured from the mucosa-associated microbiota of a healthy kitten and manufactured into a freeze-dried probiotic, we demonstrated that oral administration of *E. hirae* could mitigate the increase in intestinal permeability and fecal water loss resulting from experimental EPEC infection in purpose-bred kittens (25).

Based on these findings, we hypothesized that administration of feline-origin *E. hirae* to weaned fostered shelter kittens could provide a measurable preventative health benefit. To test this hypothesis we conducted a randomized, placebo-controlled, blinded clinical trial to determine the impact of a freeze-dried *E. hirae* probiotic on parameters of body weight gain, incidence of diarrhea requiring examination by a veterinarian, carriage of potential diarrheal pathogens, and composition of the intestinal microbiota in weaned fostered shelter kittens.

## Materials and methods

### *Enterococcus hirae* probiotic

#### Formulation

The administered freeze-dried probiotic was manufactured from a pure, well-characterized isolate of *Enterococcus hirae*. The strain (1002-2) was isolated by swab of the ileum mucosa performed during autopsy of a healthy kitten in which histological evidence of *E. hirae* attachment to the intestinal epithelium was concurrently documented (4). The isolate lacked all virulence characteristics, the results of which were previously reported (4). The selected strain was cultured for use as a probiotic in Biostat B-plus reactors continuously fed with MB medium (26) containing 20 g/L glucose. Chemostat maintained a dilution rate (*D* = 0.17 h^−1^), temperature (37°C), and pH (5.5). The viable biomass was collected from the reactor overflow, concentrated, and suspended in bacteriological peptone prior to lyophilization. Glucose consumption, accumulation of lactate and absence of alternative fermentation products were monitored with high-performance liquid chromatography (Shimadzu Corporation, Kyoto, Japan) performed under isocratic conditions at 65°C, a mobile phase of 5 mM sulfuric acid (H_2_SO_4_) at a 0.4 ml/min flow rate using an Alltech IOA-1000 organic acids column (300 mm x 7.8 mm, Alltech, IL, USA), and coupled to a refractive-index detector. Numbers of viable cells were confirmed by plating on M17 agar (27) and incubating at 37°C for 72 h. For each sample, three plates were prepared from each dilution and recorded as the average of three independent counts. Finalized freeze-dried peptone containing probiotic and vehicle (sterile ultrapure bacteriological peptone, Thermo Scientific) were stored at −80°C.

#### Compounding

Capsules containing either the probiotic or placebo (bacteriological peptone) were compounded by the NC State Veterinary Hospital Pharmacy into blue or yellow opaque capsules whose contents remained blinded to study investigators.

Pre-compounding *E. hirae* stock containing 2.24 × 10^7^ colony forming units (CFU) per milligram was stored at −80°C and transferred to dry ice when in use. A desired dose of 1 × 10^8^ CFU was achieved by compounding 4.5 mg of *E. hirae* stock with 110.5 mg of bacteriological peptone (ThermoFisher Scientific, Waltham, MA) to achieve an average pack weight of 115 mg ± 5 mg. Placebo consisted of 115 mg of bacteriological peptone only. Compounding was performed in a 4°C walk-in environmental chamber. Batch sizes of 300 capsules were compounded using a pre-chilled mortar and pestle, ProFiller 3600 capsule machine, and Letco #4 size gelatin capsules. Capsules were transferred to 30–count vials labeled with capsule color and date of manufacture and stored at −20°C.

#### Viability

Sustained numbers of *E. hirae* in the compounded probiotic was confirmed by culture of the contents of a subset of capsules immediately after formulation, after 5 months of storage at −20°C on location at the shelter, and following return of leftover (unused) capsules to the shelter after storage in the home freezer of a foster care provider. Briefly, probiotic and placebo capsule contents were dissolved separately in 1 ml of sterile phosphate-buffered saline (PBS) to generate a working stock. Serial dilutions were performed to generate concentrations ranging from 1 × 10^7^ to 1 × 10^2^ CFUs per 1 ml and the equivalent for the placebo. For each of the 10^4^, 10^3^, and 10^2^ dilutions and sterile PBS, 100 μl was spread onto three separate plates of Difco m-Enterococcus Agar (Becton Dickinson, Franklin Lakes, NJ). Plates were incubated at 37°C for 24 h. Following incubation, colonies were counted on the plates of the dilution(s) that resulted in 30 and 300 CFUs. The average colony count was calculated, allowing for the current CFU concentration of the working stock to be extrapolated. Viability of the probiotic capsules were then determined by dividing the extrapolated concentration by the initial presumed concentration of 1 × 10^8^ CFUs/ml.

### Clinical trial design

A prospective, randomized, placebo-controlled, blinded clinical study was conducted at a large municipal animal shelter [Wake County Animal Center (WCAC)] over the time interval from May 1 to October 1 in the year 2018 and 2019.

Eligibility criteria included any weaned and apparently healthy kitten aged <12 weeks and admitted to theshelter with a body weight < 0.9 kg (2 lbs) and therefore requiring temporary care prior to fitness for surgical sterilization and adoption. Kittens destined for fostering by a WCAC-trained care provider were eligible for participation in the study. Kittens that were alternatively transferred to the care of rescue partner organizations were not eligible to participate.

#### Preventative care

All kittens were housed for an average of 2–3 days in a dedicated room within the shelter prior to transfer to the home of a volunteer foster care provider. During this time, each kitten was assigned an individual identification/medical record number for tracking using a web-based shelter software program (ShelterBuddy, Wacol Brisbane QLD AUSTRALIA). Kittens were administered a single oral dose of ponazuril paste (Marquis^®^, Boehringer Ingelheim; 30mg/kg) and pyrantel pamoate (Columbia Laboratories; 11 mg/kg) and topical administration of selamectin (Revolution^®^, Zoetis) for treatment of endo- and ectoparasites. Kittens that were ≥4 weeks of age and ≥0.45 kg were vaccinated by subcutaneous injection with feline viral rhinotracheitis, calicivirus, and panleukopenia antigen (Nobivac^R^:Feline 1-HCP, Intervet INC Merck Animal Health division US Omaha NE 68103) and revaccinated every 2–3 weeks until 16–20 weeks (28).

#### Randomization

In advance of the study, each day of each month from May 1 to October 1 was randomly designated as blue or yellow using an online program (Random.org). The designation of blue or yellow indicated the treatment group to which any kittens entering foster care on that day would be assigned and was unconcealed. Participation in the study was voluntary and each foster volunteer signed an informed consent. To prevent cross-contamination of treatments, participating foster-care providers were only allowed to simultaneously foster kittens designated to the same treatment group at any given time.

#### Blinding

The placebo and probiotic were formulated into opaque size number 4 gelatin capsules colored blue and yellow to correspond with the treatment group allocation color. Study investigators, shelter staff, and foster care providers were blinded to identity of the capsule contents. Only the pharmacists (SW, EW, and GD) responsible for compounding the capsules and technician (SHS) responsible for documenting bacterial viability of capsule contents were aware of the capsule contents. All other investigators remained blinded to group allocation until all data were analyzed and results of the study were written in manuscript form.

#### Treatment and monitoring

Participating foster care providers were provided with capsules containing probiotic or placebo based on prior random allocation, two zip-lock bags for collection of a pre- and end-of-study fecal sample with labels for providing the collection date and kitten shelter identification number, observation worksheet for recording pre- and end-of-study body weight, and instructions for treatment and sample collections. Foster care providers were instructed to store the dispensed capsules in their home freezer throughout the study.

During the study, the type of diet provided to kittens was chosen at the discretion of the foster care provider. Supplements such as vitamins were permitted but not administration of non-study probiotics. Prior to beginning administration of the probiotic or placebo, foster care providers were instructed to obtain an initial body weight for which a scale (AccuWeight AW-KS001BB) was provided, and to collect a voided fecal sample with instructions to store the sample in their home freezer. After recording of an initial body weight and collection of a fecal sample, foster care providers were instructed to administer a single capsule of probiotic/placebo daily by opening the contents of the capsule onto a small portion of canned kitten food and monitoring consumption by the kitten until complete. Each foster-care provider was provided with an observation sheet for documenting daily probiotic administration and encouraged, but not required, to take periodic body weight measurements over the course of the study. Upon reaching a weight of ≥2 pounds, foster-care providers were instructed to obtain a final body weight and fecal sample while the kitten was still receiving the probiotic and store as per the initial sample. Upon completion of the study, fecal samples were transferred by the foster care provider back to Wake County Animal Center and stored at −20°C prior to transport to the laboratory on dry ice where they were subsequently stored at −80°C until analysis.

##### Incidence of diarrhea

Kittens were diagnosed as having significant diarrhea if they met criteria for unscheduled examination by the WCAC veterinary team. These criteria included diarrhea lasting more than 24 h and accompanied by straining to defecate, discomfort during defecation, or decreased appetite. Foster care providers were not trained to provide fecal scoring and fecal scores were not requested or recorded as part of the study.

### Criteria for inclusion of gathered data

Minimum criteria for inclusion in data analysis were (1) uninterrupted daily administration of the placebo/probiotic and (2) recording of a pre- and end-of-study body weight. An end-of-study body weight was not required for kittens that died or were euthanized while on the study.

### Molecular assays

#### Fecal PCR testing

Paired fecal samples, collected from kittens prior to beginning and immediately prior to completion of the study, were batched and shipped on dry ice to a collaborating laboratory (IDEXX Diagnostics Laboratory, Sacramento CA) where they were stored at −80°C prior to assay.

Fecal samples were suspended in 3 ml guanidinium thiocyanate based lysis solution (29), vortexed to facilitate organism detachment and rapid protein denaturation and incubated at room temperature for 15 min. The lysate was then used to extract total nucleic acid on a MagMax 96 Flex (Life Technologies, Valencia, CA) with magnetic beads (Roche, Indianapolis, IN) using manufacturer's guidelines. Total nucleic acid was eluted in 200 μl of PCR-grade nuclease-free water and 5 μl amplified in subsequent single plex real-time PCR reactions.

Real-time PCR was performed with proprietary forward and reverse primers and hydrolysis probes. Target genes for enteropathogen detection were as follows: Feline enteric coronavirus 7b gene (DQ010921.1), Feline panleukopenia virus VP2 gene (EU252145), Rotavirus VP7 gene (EU708950), *Salmonella enterica* invasion A gene (EU348366), *Campylobacter* (*coli and jejuni*) IpxA gene [AY531496 (*C. coli*) and AL111168 (*C. jejuni*)], *Clostridium perfringens* alpha toxin gene (L43545), *Clostridium perfringens* enterotoxin gene (X81849), *Shigella* spp. RFC gene (AE005674), *E. coli* virulence factor genes (F4 (K88), F5 (K99), F6 (987P) and F18ab fimbrial adhesin genes; STX2e gene; Intimin adhesion gene; LT1 gene, ST1a and ST1b genes) (EU570252; M35282; M35257; GQ325633; GU945540; ECU38618; S60731.1; M34916), *Yersinia* spp. invasion locus protein (ail) gene (AJ605740), *Cryptosporidium* spp. ssrRNA gene (A093489), *Giardia* small-subunit rRNA gene (DQ836339), *Toxoplasma gondii* internal transcribed spacer-1 gene (L49390, *Tritrichomonas foetus* 5.8S rRNA gene (AF339736), *Blastocystis* spp. SSU rRNA gene (AB023499; KP890050; AB070992; AB071000; AB070999; AB070990; AB070991; AB107971; AF408426), *Toxocaris leoni* ITS-2 gene (Y09490), *Toxocara* (*malayasiensis and cati*) ITS-1 gene, Internal transcribed spacer-2 (ITS-2) gene (AB110033; AM231609), *Ancylostoma* spp. ITS-1 gene (DQ438074; DQ438063; DQ780009), *Trichuris* spp. ITS-2 gene (AM234616), *Uncinaria* spp. ITS-1 gene (AF217890), *Parascaris* spp. ITS-1 gene (AJ007459), *Echinococcus* spp. rRNA between the Cox 1 and Cox 2 genes (NC_000928), *Pythium* spp. ITS-1 gene (EF016907.1), and *Prototheca* spp. ITS-1 gene (AJ245645).

Real-time PCR was run with six quality controls as previously described (30) including (1) PCR positive controls [synthetic DNA (Integrated DNA Technologies IDT, Coralville, IA), run quantitatively], (2) PCR negative controls (RNase-free PCR-grade water, Fisher Scientific, Waltham MA), (3) negative extraction controls (lysis solution only), (4) DNA pre-analytical quality control targeting mammalian ssr rRNA (18S rRNA) gene complex, (5) environmental contamination monitoring control (swab-based laboratory monitoring), and (6) spike-in internal positive control. These controls assessed the functionality of the PCR test protocols for the (1), functional assessment of the real-time PCR test performance (2) absence of contamination (both PCR product carry-over and sample cross-contamination), (3) absence of detectable cross-contamination during the extraction process, (4) quality and integrity of the DNA as a measure of sample validity (by quantitatively assessing 18S gene load), reverse transcription protocol (5) absence of aerosol-based contamination within the PCR laboratory space, and (6) absence of PCR inhibitory substances as a carryover from the sample matrix (internal sample control with spike-in DNA).

Analysis was performed on a Roche LightCycler 480 (Roche Applied Science, Indianapolis (IN) on which amplification data were analyzed using the 2nd derivative maximum method to generate crossing points (Cp values). Each Cp value was quantitatively validated by prior calibration to a standard curve of DNA for each target assay.

#### 16S rRNA gene amplicon sequencing

Fecal samples that were collected immediately prior to completion of the study were selected for microbiota analysis. To isolate the impact of the probiotic intervention and minimize bias in the data, sibling kittens had only a single/representative fecal sample chosen for analysis.

DNA isolation from fecal samples was performed as previously described (31). Briefly, 0.3 ml of Qiagen ATL buffer (Valencia, CA) supplemented with 60 mg/ml lysozyme (Thermo Fisher Scientific, Grand Island, NY) was added to the samples. The suspensions were transferred to 2 ml tubes containing 106/500 μm glass beads (Sigma, St. Louis, MO) and incubated for 1 h at 37°C with occasional agitation. The suspensions were agitated for 40 min on a Digital Vortex Mixer. Subsequently, the suspensions were supplemented with 20 μL of Qiagen proteinase K and 0.3 ml of Qiagen AL buffer and incubated at 55°C overnight. After brief centrifugation, supernatants were aspirated and transferred to a new tube containing 0.3 ml of ethanol. DNA was purified using a standard on-column purification method with Qiagen buffers AW1 and AW2 as washing agents and eluted in 27.5 μl of DNase free water and quantified using QuantIT^®^ PicoGreen^®^.

Extracted DNA was quantified *via* PicoGreen analysis and used for bacterial 16S rRNA gene amplicon sequencing. Total DNA (12.5 ng) was amplified using universal primers targeting the V4 region of the bacterial 16S rRNA gene (32). The sequences of the primers were: 515F−5′ TCGTCGGCAGCGTCAGATGTGTATAAGAGACAGGTGCCAGCMGCCGCGGTAA 3′ and 806R−5′GTCTCGTGGGCTCGGAGATGTGTATAAGAGACAGG GACTACHVGGGTWTCTAAT 3′. Overhang adapters were appended to the 5′ end of each primer sequence for compatibility with the Illumina sequencing platform. Master mixes contained 12.5 ng of total DNA, 0.2 μM of each primer and 2× KAPA HiFi HotStart ReadyMix (KAPA Biosystems, Wilmington, MA). The thermal profile for the amplification of each sample had an initial denaturing step at 95°C for 3 min, followed by 25 cycles of denaturing of 95°C for 30 s, annealing at 55°C for 30 s for 16S rRNA and a 30 s extension at 72°C, a 5 min extension at 72°C and a final hold at 4°C. Each 16S amplicon was purified using the AMPure XP reagent (Beckman Coulter, Indianapolis, IN). In the next step each sample was amplified using a limited cycle PCR program, adding Illumina sequencing adapters and dual index barcodes [index 1(i7) and index 2(i5)] (Illumina, San Diego, CA) to the amplicon target. The thermal profile for the amplification of each sample had an initial denaturing step at 95°C for 3 min, followed by a denaturing cycle of 95°C for 30 s, annealing at 55°C for 30 s and a 30 second extension at 72°C (8 cycles), a 5 min extension at 72°C and a final hold at 4°C. The final libraries were again purified using the AMPure XP reagent (Beckman Coulter), quantified with Quant-iT™ PicoGreen^®^ dsDNA Reagent (Molecular Probes, Thermo Fisher Scientific, Waltham, MA), and normalized prior to equimolar pooling. The DNA library pool was then denatured with NaOH, diluted with hybridization buffer and heat denatured before loading on the MiSeq reagent cartridge MiSeq instrument (Illumina). Automated cluster generation and paired–end sequencing with dual reads were performed according to the manufacturer's instructions.

### Data analysis

Kitten populations were described in terms of age, sex, weight on intake to the shelter, number of kittens in litter, time spent on study, average daily gain in body weight, weight on exit from study, episodes of diarrhea requiring examination by shelter veterinary staff, and outcome (alive–vs.–died or euthanized). Categorical data (e.g., proportions) were tested for significant differences between treatment groups using a Chi-square with calculation of odds ratios and 95% confidence intervals. Continuous data were tested for normal distribution and equal variance followed by parametric (one-way ANOVA) or non-parametric (Kruskal-Wallis one-way ANOVA on ranks) analysis as appropriate.

The proportion of kittens testing PCR positive for detection of selected infectious agents in feces were compared for significant differences between pre- and post-study samples within each treatment group using a Chi Square test or Fisher Exact test. Infectious agents for which ≤5 kittens tested positive at both time points were not subjected to statistical analysis. The median burden of selected infectious agent RNA/DNA detected within feces of test-positive kittens, as represented by the PCR Cp values, was compared for significant differences between pre- and post-study samples within each treatment group using a Kruskal-Wallis one-way ANOVA on ranks. Testing was performed using Systat software (SigmaPlot 12).

Sequencing output from the Illumina MiSeq platform were converted to fastq format and demultiplexed using Illumina Bcl2Fastq 2.18.0.12. The resulting paired-end reads were processed using QIIME 2 (33) 2018.11. Index and linker primer sequences were trimmed using the QIIME 2 invocation of cutadapt. The resulting paired-end reads were processed with DADA2 through QIIME 2 including merging paired ends, quality filtering, error correction, and chimera detection. Amplicon sequencing units from DADA2 were assigned taxonomic identifiers with respect to Green Genes release 13_08.

Alpha diversity estimates were calculated within QIIME 2 using Evenness (Shannon) index and observed species number metrics at a rarefaction depth of 5,000 reads. Pairwise significance was tested using a Kruskal-Wallis ANOVA with Benjamini-Hochberg corrected q-values calculated as implemented in QIIME 2. Beta diversity estimates were calculated within QIIME 2 using weighted and unweighted Unifrac distances as well as Bray-Curtis dissimilarity between samples at a subsampling depth of 5,000 reads. Results were summarized, visualized through principal coordinate analysis in Emperor, and significance was estimated by PERMANOVA with Benjamini-Hochberg corrected q-values calculated as implemented in QIIME 2. Relative abundance data were normalized by sample library size and taxa were removed if they were present in <10% of all samples or if lower than 0.01% average abundance (34). Differences in abundance were tested for significance using a Kruskal-Wallis One-Way ANOVA on Ranks. When significant, pairwise comparisons between groups were performed using a Dunn's test. Generated *p-*values were corrected for multiple testing using the Benjamini-Hochberg procedure (35) at a false discovery rate of 0.25. For selected taxa, odds ratio and 95% confidence intervals for association with treatment group were calculated using a Chi-square Test with Yates Continuity Correction. Statistical examination of abundance data was conducted using Systat Software (SigmaPlot 12.0, San Jose, CA). Results were represented graphically using GraphPad Software (Prism version 7.03, San Diego, CA).

## Results

### Population description

**Figure 1:**
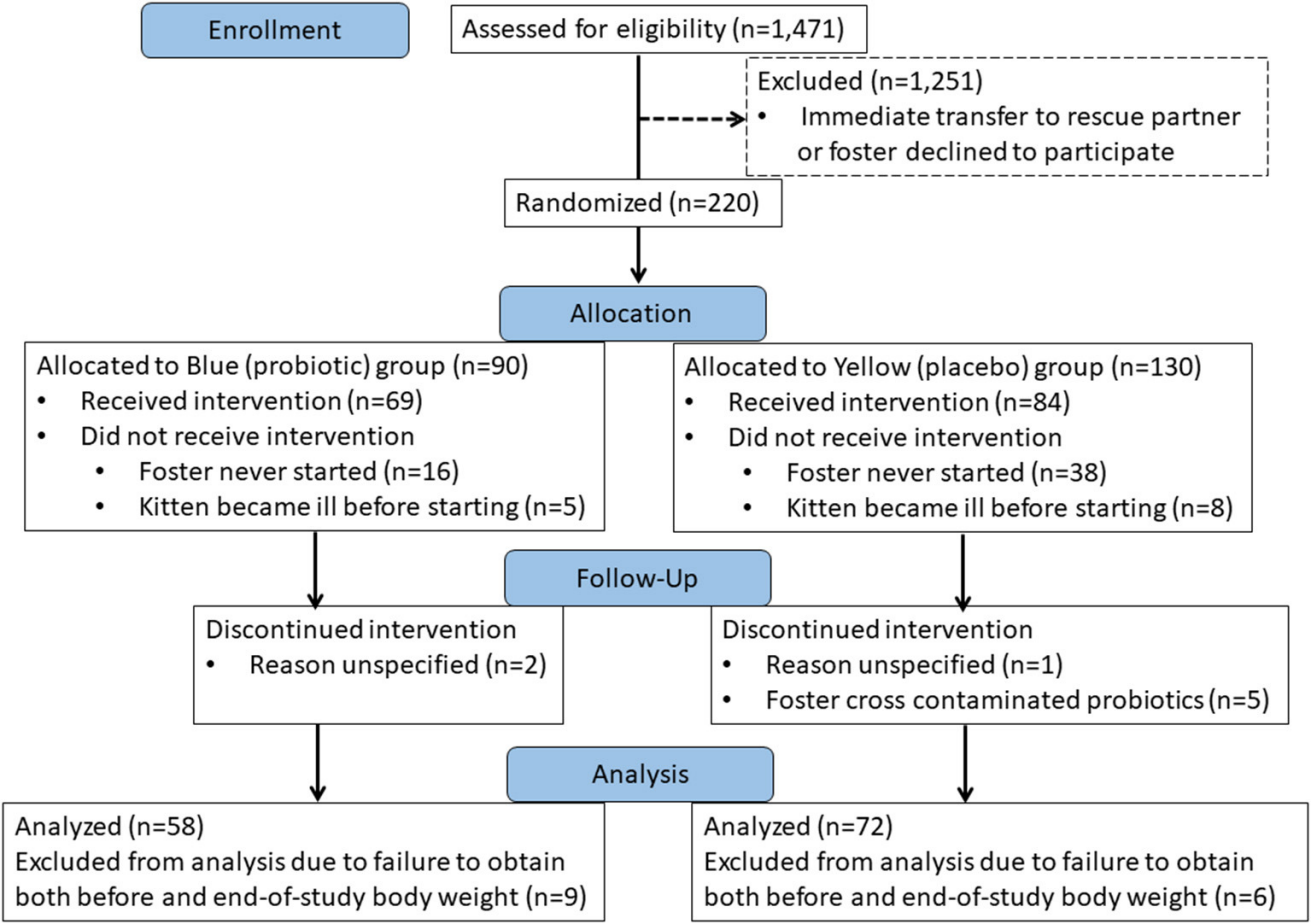
CONSORT Flow diagram showing disposition of kittens over the course of the study.

Over the time interval from May 1 to October 1 in the years 2018 and 2019, a total of 1,471 kittens were assessed for eligibility to participate in the study. Among these kittens, 220 (15%) were randomly allocated to treatment with probiotic or placebo. One-hundred thirty kittens completed the study. Fifty-eight kittens from 40 unrelated litters received the probiotic (blue group) and 72 kittens from 37 unrelated litters received the placebo (yellow group) (Figure 1). A total of 28 different foster care providers participated over the 2-year course of the study, each fostering a median of 3 kittens (range, 1–18 kittens).

### Clinical outcome

**Figure 2:**
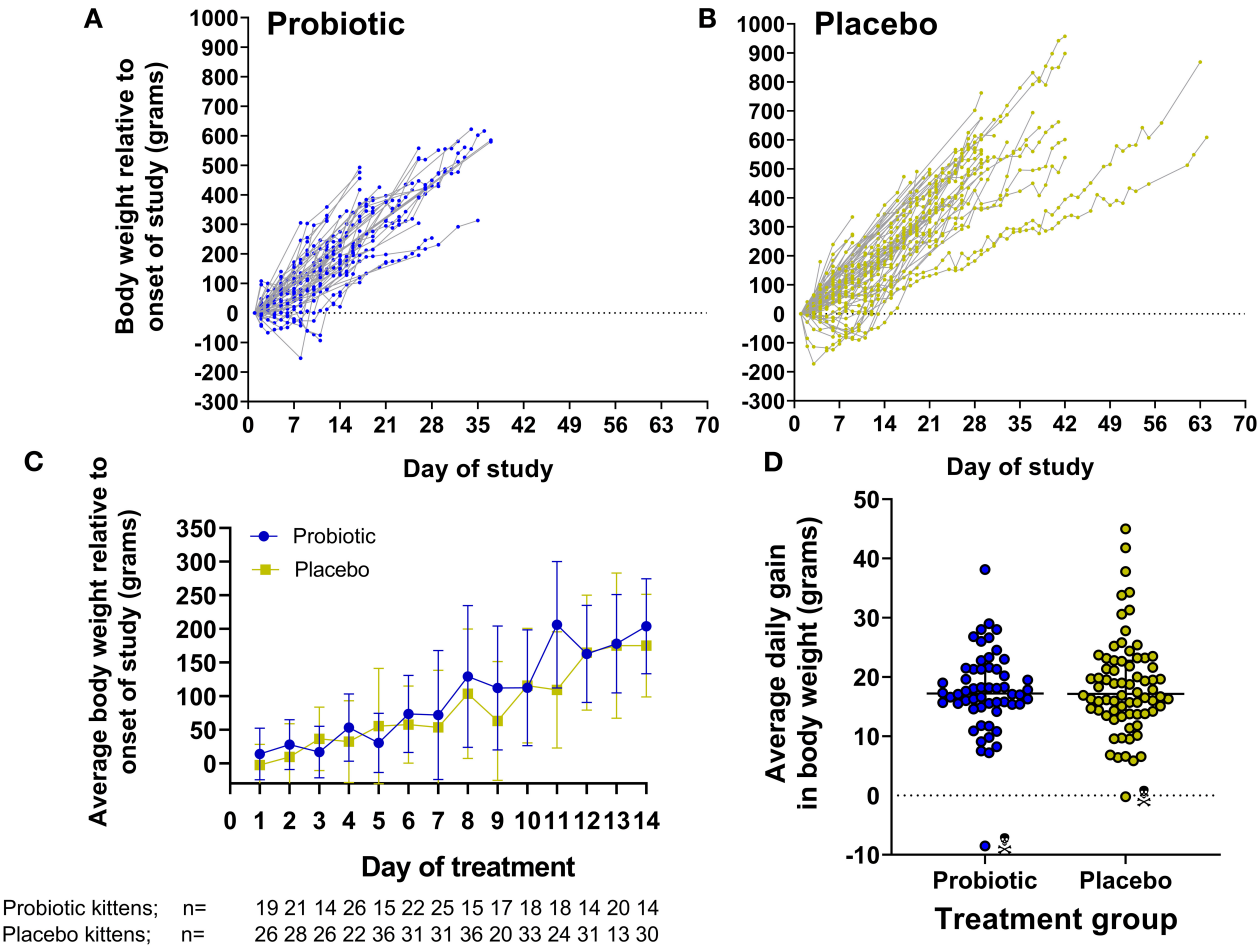
Gain in body weight by kittens receiving probiotic or placebo. **(A,B)** Show daily gain in body weight normalized by initial body weight for individual kittens in each group over the course of the study. **(C)** Represents the average ± SD body weight of kittens comparing both groups on each day during the first 14 days of the study. Note that each time point represents only those kittens having their body weight measured on the given day which is indicated below the x-axis legend. **(D)** Demonstrates the average daily gain in body weight over the course of the study [(end weight – start weight)/number of days between weight measurements] in individual kittens comparing both groups. Kittens that died/where euthanized are designated by skull symbol.

Viability of the probiotic (target dose, 1 × 10^8^ CFU) was not diminished when quantified after storage at −20°C at the shelter (2.2–8.9 × 10^8^ CFU), nor followed by storage in the home freezer of a foster care provider (0.94 × 10^8^ CFU). All kittens readily consumed the probiotic or placebo when offered. When comparing the probiotic and placebo groups, there were no significant differences in starting age, weight upon initiation of the study, number of days in the study, average daily gain in body weight, or weight at completion of the study (Table 1 and Figure 2).

**Table 1:** Population description of 130 kittens treated with probiotic or placebo.

**Parameters evaluated**	**Probiotic (*n* = 58) male = 32, female = 26 undetermined = 0**		**Placebo (*n* = 72) male = 36, female = 35, undetermined = 1**		**Kruskal-Wallis One-Way ANOVA on Ranks *p*-value**
	**Median**	**IQR**	**Median**	**IQR**	
Estimated age (weeks)	6	5–8	6	5–7	0.429
Number of days in study	17	14–26	22	14–31	0.123
Weight at start of study (grams)	602	455–704	501	414–649	0.075
Weight at end of study (grams)	933^†^	883–998	964	908–1,021	0.205
Average daily gain in body weight (grams/day)^a^	17^†^	15–21	17	14–23	0.904
	**No**.	**%**	**No**.	**%**	**Chi-square test *p-* value**
Kittens examined for diarrheal illness	6^b^	10	20^c^	28	0.02
Kittens euthanized for diarrheal illness	0	0	0	0	–
Kittens euthanized for non-diarrheal illness	3	5	1	1	0.17
Kittens that survived	55	95	71	99	

IQR, interquartile range.

a Calculated as = (end weight–start weight)/number of days between weight measurements.

b From 6 different litters.

c From 8 different litters.

† Excluding 2 kittens that were euthanized.

Kittens treated with the probiotic were 3.4 times less likely to develop diarrhea compared to kittens treated with the placebo (odds ratio = 0.294, 95% CI 0.109–0.792, *p* = 0.022). No kittens were euthanized due to diarrheal illness and overall survival was not significantly different between the 2 groups. Reasons for euthanasia of kittens in the study included failure to thrive (2 probiotic kittens), panleukopenia (1 probiotic kitten), and major medical (1 placebo kitten) (Table 1).

### Prevalence and abundance of selected infectious agents

Quantitative PCR was performed on paired fecal samples, collected prior to beginning and immediately prior to completion of the study, from 37 kittens receiving the probiotic and 55 kittens receiving placebo. Reasons for exclusion of kittens were lack of either a pre or post-fecal sample (13 probiotic kittens, 15 placebo kittens), insufficient feces for both microbiome and PCR analysis (5 probiotic kittens, 1 placebo kitten), or euthanasia prior to final fecal sample collection (3 probiotic kittens, 1 placebo kitten).

Fecal samples were tested for the presence of 30 different bacterial, viral, protozoal, fungal, algal, and parasitic agents, among which 17 were detected in ≥1 kitten (Tables 2 and 3). Prior to initiation of the study, 92% (34/37) of kittens receiving probiotic and 87% (48/55) of kittens receiving placebo had a median of 2 infectious agents (range, 0–4) identified. Upon completion of the study, 86% (32/37) of kittens receiving probiotic and 78% (43/55) of kittens receiving placebo had a median of 2 (range, 0–4) and 1 (range, 0–4) respectively, infectious agents identified. The number of identified agents did not differ significantly from pre-study values in either group. Examination of individual kittens demonstrated that 22% (8/37) of kittens receiving the probiotic had a median gain or loss in number of infectious agents of 0 (range, −3 to +3) and 20% (11/55) of kittens receiving the placebo had a median gain or loss in number of infectious agents of 0 (range, −4 to +3) which did not differ significantly between the groups.

**Table 2:** Prevalence of infectious agents identified by RT-PCR or PCR in RNA/DNA extracted from voided feces collected from fostered kittens prior to (pre-study) and upon completion (post-study) of ongoing daily treatment with a probiotic or placebo.

**Selected agents**	**Prevalence/presence of infectious agent(s)**													
	**Probiotic (*n* = 37)**							**Placebo (*n* = 55)**						
	**Pre-study**			**Post-study**			***P*-value**	**Pre-study**			**Post-study**			***P*-value**
	**# kittens passing QC**	**# kittens positive**	**% kittens positive**	**# kittens passing QC**	**# kittens positive**	**% kittens positive**		**# kittens passing QC**	**# kittens positive**	**% kittens positive**	**# kittens passing QC**	**# kittens positive**	**% kittens positive**	
*Coronavirus*	37	16	43.2	37	14	37.8	0.813	55	20	36.4	55	17	30.9	0.686
*E.coli* Intimin (*eae*)	34	10	29.4	33	6	18.2	0.429	53	8	15.1	48	7	14.6	0.835
Feline *Panleukopenia*	34	9	26.5	33	2^†^	6.1	0.054	53	10	18.9	44	0	0.0	**0.002** ^†^ ^†^
*Salmonella* spp.	34	0	0.0	33	2	6.1	–	52	4	7.7	44	6	13.6	0.505
*Cryptosporidium* spp.	34	2	5.9	33	0	0.0	–	53	3	5.7	44	3	6.8	–
*Giardia* spp.	34	0	0.0	33	0	0.0	–	52	0	0.0	45	3	6.7	–
*Toxoplasma gondii*	34	0	0.0	33	1	3.0	–	52	0	0.0	44	0	0.0	–
Campylobacter jejuni	34	0	0.0	33	2	6.1	–	53	2	3.8	45	2	4.4	–
*Campylobacter campi*	34	0	0.0	33	2	6.1	–	52	0	0.0	45	2	4.4	–
*Toxocaris leoni*	34	1	2.9	33	0	0.0	–	52	0	0.0	44	0	0.0	–
*Toxocara malayasiensis*	34	1	2.9	33	0	0.0	–	52	0	0.0	44	1	2.3	–
*Toxocara cati*	34	1	2.9	33	1	3.0	–	52	0	0.0	44	1	2.3	–
*Ancylostoma* spp.	34	0	0.0	33	0	0.0	–	52	1	1.9	44	0	0.0	–
*Pythium* spp.	35	2	5.7	33	3	9.1	–	52	2	3.8	45	3	6.7	–
*Shigella* spp.	34	0	0.0	33	0	0.0	–	52	4	7.7	44	0	0.0	–
*Trichuris* sp.	34	0	0.0	33	0	0.0	–	52	0	0.0	45	1	2.2	–
*Clostridium perfringens* Alpha toxin	35	28	80	35	28	80	0.765	55	44	80	48	30	62.5	0.080
> 300 Thous/g feces	35	6	17.1	35	2	5.7	0.259	55	7	12.7	48	5	10.4	0.955
*Clostridium perfringens* Enterotoxin	34	3	8.8	33	1	3.0	–	52	3	5.8	44	0	0	–
> 300 Thous/g feces	34	1	2.9	33	0	0	–	52	0	0	44	0	0	–

QC, Quality Control. The proportion of kittens testing PCR positive for detection of selected infectious agents in feces were compared for significant differences between pre- and post-study samples within each treatment group using a Chi Square or Fischer Exact test.

– Infectious agents for which ≤ 5 kittens tested positive at both time points were not subjected to statistical analysis.

† Both kittens negative for panleukopenia DNA at time of pre-study PCR testing.

†† Remains significant after correction for multiple testing using a Benjamini-Hochberg false discovery rate of 0.15.

**Table 3:** Abundance of infectious agents identified by RT-PCR or PCR in RNA/DNA extracted from voided feces collected from fostered kittens prior to (pre-study) and upon completion (post-study) of ongoing daily treatment with a probiotic or placebo.

**Selected agents**	**Burden of infectious agent(s)**													
	**Probiotic (*n* = 37)**							**Placebo (*n* = 55)**						
	**Pre-study**			**Post-study**			**Kruskal-Wallis ANOVA**	**Pre-study**			**Post-study**			**Kruskal-Wallis ANOVA**
	**Median** **Cp**	**Min** **Cp**	**Max** **Cp**	**Median** **Cp**	**Min** **Cp**	**Max** **Cp**		**Median** **Cp**	**Min** **Cp**	**Max** **Cp**	**Median Cp**	**Min** **Cp**	**Max** **Cp**	
*Coronavirus*	38	23	39	29	23	39	0.151	38	21	39	28	24	39	0.035^†^
*E.coli* Intimin (*eae*)	34	29	37	35	26	36	1.00	32	20	36	35	26	38	0.769
Feline *Panleukopenia*	20	17	25	20	15	26	1.00	22	16	24	–	–	–	–
*Salmonella* spp.	–	–	–	40	39	40	–	39	37	40	38	36	38	0.352
*Cryptosporidium* spp.	37	37	37	–	–	–	–	38	36	38	38	36	38	–
*Giardia* spp.	–	–	–	–	–	–	–	–	–	–	35	34	40	–
*Toxoplasma gondii*	–	–	–	37	37	37	–	–	–	–	–	–	–	–
Campylobacter jejuni	–	–	–	38	38	38	–	30	28	32	38	37	39	–
*Campylobacter campi*	–	–	–	35	34	37	–	–	–	–	36	36	36	–
*Toxocaris leoni*	37	37	37	–	–	–	–	–	–	–	–	–	–	–
*Toxocara malayasiensis*	36	36	36	–	–	–	–	–	–	–	34	34	34	–
*Toxocara cati*	38	38	38	35	35	35	–	–	–	–	36	36	36	–
*Ancylostoma* spp.	–	–	–	–	–	–	–	36	36	36	–	–	–	–
*Pythium* spp.	39	38	40	38	37	39	–	39	39	40	39	38	39	–
*Shigella* spp.	–	–	–	–	–	–	–	34	30	37	–	–	–	–
*Trichuris* sp.	–	–	–	–	–	–	–	–	–	–	39	39	39	–
*Clostridium perfringens* Alpha toxin	31	22	35	31	27	34	0.164	30	22	35	32	23	35	0.186
gene copies/ g feces	51,310	3,335	23,235,026	35,044	4,160	465,721	0.164	89,716	2,601	20,236,834	26,222	3,200	8,652,445	0.186
*Clostridium perfringens* Enterotoxin	29	25	31	29	29	29	–	33	32	33	0	0	0	–
gene copies/g feces	109,174	36,401	2,547,614	157,442	157,442	157,442	–	12,827	10,212	17,624	0	0	0	–

Cp, PCR cycle threshold. – Infectious agent not detected.

† Not significant after correction for multiple testing using a Benjamini-Hochberg false discovery rate of 0.15.

The most common infectious agents having RNA or DNA demonstrated in feces were feline enteric coronavirus, attaching and effacing *E. coli* (presence of *eae*), panleukopenia, and *Clostridium perfringens* (alpha-toxin). There were no significant differences in prevalence (Table 2) or abundance (Table 3) of infectious agents between the 2 groups of kittens for fecal samples collected prior to initiation of the study. Upon completion of the study there was a significant decrease in prevalence of detection of panleukopenia DNA in kittens treated with the placebo (Table 2). Decrease in abundance of coronavirus RNA in kittens treated with placebo was not significant after correction for multiple comparisons (Table 3).

Infectious agent PCR testing was performed on feces collected from 16/26 kittens that developed diarrhea over the course of the study. Identified infectious agents having a potential to contribute to diarrhea included panleukopenia (4 kittens from 2 litters), feline enteric coronavirus (5 kittens from 3 litters), *Campylobacter* spp. (3 kittens from 2 litters), attaching and effacing *E. coli* (2 kittens), and alpha-toxin positive *Clostridium perfringens* (alpha toxin gene copies >30,000/gram feces) (2 kittens).

### Microbial community composition

Analysis of the fecal microbiota by 16S rRNA gene amplicon sequencing was performed for 32/58 (55%) unrelated kittens that received the probiotic (32/40 (80%) of litters represented) and 35/72 (49%) unrelated kittens that received the placebo (35/37 (94%) of litters represented). Included in the analysis were 6 kittens from 6 individual litters that received probiotic and were examined for diarrheal illness during the course of the study. Eight litters in the probiotic group did not have a kitten represented in the analysis due to lack of an end-of-study fecal sample (5 litters) or euthanasia (3 litters). Also included in the analysis were 7 kittens from 7 individual litters that received placebo and were examined for diarrheal illness during the course of the study. Two litters in the placebo group did not have a kitten represented in the analysis due to lack of an end-of-study fecal sample (one of which included a kitten that developed diarrhea during the course of the study).

**Figure 3:**
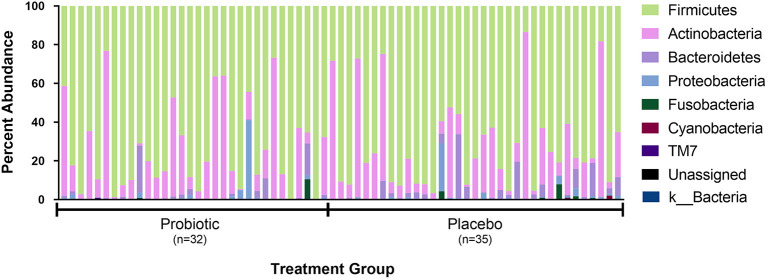
Percent abundance of the major phyla observed in feces from 67 individual kittens. Each sample is designated as having been obtained from a kitten receiving either the probiotic or placebo. Not shown in legend are phyla observed in <10 kittens and at an abundance <0.15% in all samples (i.e., Acidobacteria, Chlamydiae, Chlorobi, Chlorflexi, Gemmatimonadetes, Tenericutes, and Verrucomicrobia).

**Figure 4:**
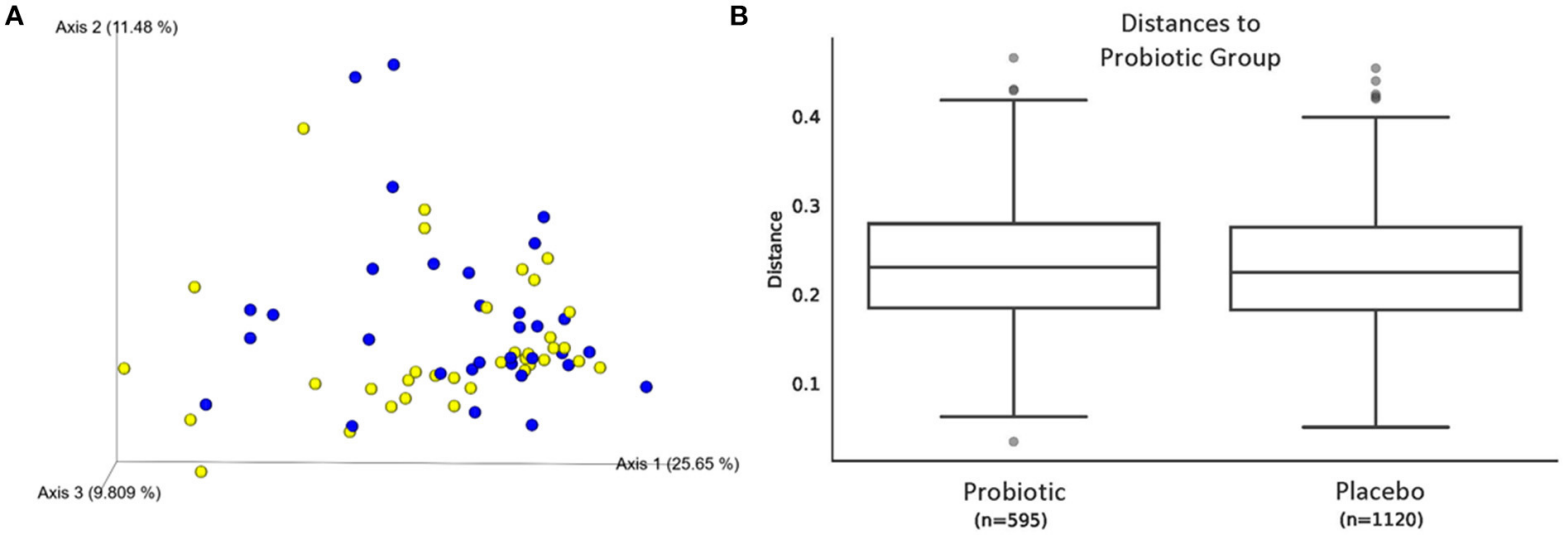
Beta diversity of microbiota from 67 kittens receiving probiotic or placebo. **(A)** Weighted UniFrac principal coordinates analysis plot showing similar phylogenic diversity of microbial communities from feces of kittens receiving the probiotic (blue circles) or placebo (yellow circles). Axis 1, 25.65%; Axis 2, 11.48%; Axis 3, 9.809%. **(B)** Weighted Unifrac distances between microbial communities shown in **(A)**. PERMANOVA *q* = 0.755.

The sequence analysis of all 67 kittens yielded 5,675,488 quality sequences (mean ± SD = 84,709 ± 34,177). The major phyla represented in the fecal microbiota of kittens were *Firmicutes, Actinobacteria, Bacteroidetes, Proteobacteria*, and *Fusobacteria* (Figure 3). There were no significant differences in alpha diversity measures of evenness (Shannon), diversity (Faith), or observed number of taxa between kittens receiving the probiotic compared to placebo. Both weighted and unweighted measures of beta diversity did not identify significant differences in phylogenetic composition of the microbiota between kittens receiving the probiotic vs. placebo (Figure 4).

**Figure 5:**
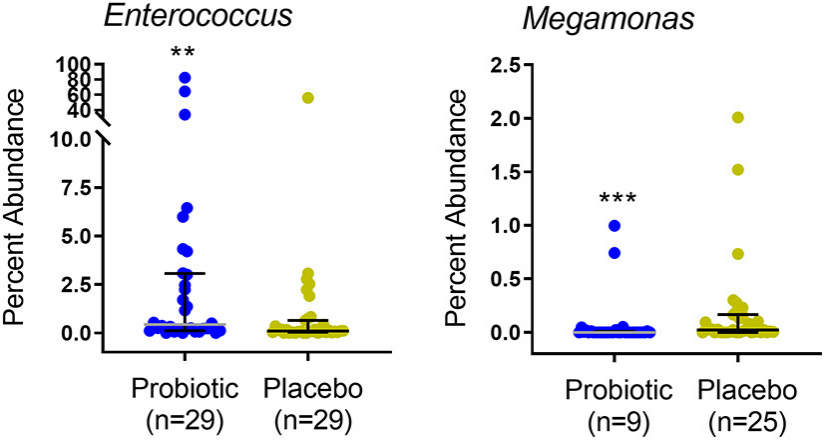
Percent abundance of *Enterococcus* and *Megamonas* in feces of 67 kittens receiving probiotic (32 kittens, blue circles) or placebo (35 kittens, yellow circles). Data points represent individual kittens. Bars represent median and interquartile range. *n* = number of kittens having the OTU at an abundance >0. Kruskal-Wallis ANOVA **p = 0.005 (BH-corrected *p* = 0.24). ***p < 0.001 (BH-corrected *p* < 0.09). BH, Benjamini-Hochberg.

Kittens receiving the probiotic had a significantly higher median relative abundance of the genus *Enterococcus* and lower abundance and prevalence of *Megamonas* (Figure 5). Abundance of specific taxa observed in the feces of kittens receiving probiotic vs. placebo are shown in Table 4. Kittens receiving the probiotic were 10.9 times less likely to have detectable *Megamonas* (odds ratio = 0.157; 95%CI = 0.054–0.453, *p* < 0.001, Chi-square test) present in the microbial community compared to kittens receiving the placebo.

**Table 4:** Relative abundance and prevalence of taxa (percent of total sequences) observed by amplification and sequencing of 16S rDNA from feces of 67 kittens randomized to treatment with daily *E. hirae* probiotic (32 kittens) or placebo (35 kittens).

**Phylum**	**Family**	**Genus**	**Relative % abundance**					**Prevalence among kittens**				
			**Probiotic (*****n** **=*** **32 kittens)**		**Placebo (*****n** **=*** **35 kittens)**		**Kruskal-Wallis ANOVA *p-*value**	**Probiotic (*****n** **=*** **32 kittens)**		**Placebo (*****n** **=*** **35 kittens)**		**Chi-square *p-*value**
			**Median**	**Range**	**Median**	**Range**		**#**	**%**	**#**	**%**	
Unassigned			0.0022	0–0.44	0.0	0–0.230	0.152	18	56.3	12	34.3	0.119
Bacteria			0.012	0–0.15	0.016	0–0.52	0.234	23	71.9	27	77.1	0.831
Actinobacteria			12.9	0.043–76.0	10.9	1.08–86.3	0.851	32	100	35	100	–
	o__Actinomycetales		0.0	0–0.028	0.0	0–0.052	0.750	3	9.38	4	11.4	1.00
	Actinomycetaceae	Actinomyces	0.0	0–0.087	0.0	0–0.041	0.039	15	46.9	7	20	0.038
	Corynebacteriaceae	Corynebacterium	0.0	0–0.015	0.0	0–0.20	0.229	4	12.5	8	22.9	0.432
	Bifidobacteriaceae	Bifidobacterium	0.34	0–66.1	0.40	0–52.4	0.702	29	90.6	34	97.1	0.342
	Coriobacteriaceae		0.40	0–63.1	0.16	0–5.59	0.129	28	87.5	32	91.4	0.701
		Adlercreutzia	0.022	0–1.26	0.057	0–2.88	0.381	19	59.4	23	65.7	0.777
		Collinsella	6.7	0–33.0	8.46	0.75–74.9	0.16	31	96.9	35	100	0.478
		Slackia	0.17	0–1.31	0.27	0–1.62	0.429	29	90.6	33	94.3	0.664
Bacteroidetes			0.43	0–24.3	1.12	0.012–33.1	0.132	31	96.9	35	100	0.478
	Bacteroidaceae	Bacteroides	0.031	0–23.3	0.16	0–18.5	0.097	26	81.3	31	88.6	0.501
	Porphyromonadaceae	Parabacteroides	0.0	0–1.0	0.0	0–4.08	0.021	6	18.8	16	45.7	0.037
	Prevotellaceae	Prevotella	0.19	0–10.3	0.21	0–13.7	0.372	23	71.9	26	74.3	0.957
		[Prevotella]	0.0	0–2.1	0.0	0–1.53	0.557	4	12.5	6	17.1	0.736
Cyanobacteria			0.0	0–0.087	0.0	0–2.06	0.173	4	12.5	9	25.7	0.291
	o__Stramenopiles		0.0	0–0.087	0.0	0–0.0	0.865	4	12.5	5	14.3	1.00
Firmicutes			81.4	23.2–99.8	78.7	13.5–96.6	0.53	32	100	35	100	–
	o__Bacillales		0.00	0–0.0	0.0	0–0.14	0.105	1	3.13	5	14.3	0.200
	Bacillaceae		0.015	0–0.26	0.0086	0–0.76	0.726	19	59.4	21	60	0.844
	Bacillaceae	Anaerobacillus	0.0	0–0.12	0.0	0–0.20	0.466	9	28.1	11	31.4	0.978
		Anoxybacillus	0.0	0–0.19	0.0	0–0.61	0.525	9	28.1	11	31.4	0.978
		Staphylococcus	0.0	0–0.0	0.0	0–0.25	0.090	4	12.5	10	28.6	0.188
	Enterococcaceae		0.0	0–0.17	0.0	0–0.38	0.502	8	25	6	17.1	0.625
		Enterococcus	0.43	0–82.5	0.10	0–56.0	**0.005** ^†^	29	90.6	29	82.9	0.480
		Lactobacillus	0.058	0–26.5	0.014	0–69.6	0.115	23	71.9	20	57.1	0.317
	Streptococcaceae	Lactococcus	0.0	0–0.29	0.0	0–0.57	0.283	10	31.3	6	17.1	0.286
		Streptococcus	0.020	0–2.5	0.0	0–33.2	0.099	23	71.9	17	48.6	0.090
	Turicibacteraceae	Turicibacter	0.0	0–0.13	0.0	0–4.86	0.372	9	28.1	12	34.3	0.780
	o__Clostridiales		0.0	0–1.3	0.0052	0–0.47	0.064	9	28.1	19	54.3	0.055
	o__Clostridiales		0.32	0–10.1	0.15	0–14.9	0.900	28	87.5	29	82.9	0.736
	Clostridiaceae		0.0	0–0.0	0.0	0–0.20	0.028	0	0.0	5	14.3	0.054
		Candidatus Arthromitus	0.0	0–0.42	0.0	0–0.57	0.618	8	25	10	28.6	0.957
		Clostridium	9.50	0.22–77.5	13.0	0.40–83.2	0.280	32	100	35	100	–
		Pseudoramibacter Eubacterium	0.0	0–0.094	0.0	0–0.051	0.051	11	34.4	5	14.3	0.101
	Lachnospiraceae		0.031	0–1.06	0.024	0–0.77	0.617	20	62.5	24	68.6	0.791
	Lachnospiraceae		1.15	0.033–31.2	0.99	0.036–14.6	0.930	32	100	35	100	–
		Blautia	7.57	0.29–35.0	7.82	0.20–30.8	0.950	32	100	35	100	–
		Clostridium	1.33	0.39–50.1	0.29	0–59.9	0.023	32	100	32	91.4	0.240
		Coprococcus	0.13	0–2.4	0.16	0–2.3	0.850	30	93.8	28	80	0.153
		Dorea	0.87	0–15.1	1.02	0.015–15.1	0.782	31	96.9	35	100	0.478
		Epulopiscium	0.0	0–0.27	0.0	0–2.40	0.356	9	28.1	12	34.3	0.780
		Hespellia	0.0	0–0.081	0.0	0–0.085	0.148	8	25	4	11.4	0.259
		Roseburia	0.034	0–1.44	0.0061	0–2.3	0.050	24	75	18	51.4	0.082
		[Ruminococcus]	1.68	0.97–15.4	1.56	0.027–9.9	0.821	32	100	35	100	–
	Peptococcaceae	Peptococcus	0.023	0–3.5	0.0019	0–6.07	0.837	17	53.1	18	51.4	0.916
	Peptostreptococcaceae		0.0082	0–0.49	0.0	0–0.53	0.357	17	53.1	14	40	0.406
	Peptostreptococcaceae		1.37	0.11–82.5	0.50	0–42.7	0.380	32	100	34	97.1	1.00
		Peptostreptococcus	0.0	0–0.63	0.0	0–0.12	0.723	9	28.1	9	25.7	0.957
	Ruminococcaceae		0.13	0–6.46	0.20	0–6.5	0.518	29	90.6	32	91.4	1.00
	Ruminococcaceae		0.052	0–5.49	0.18	0–4.3	0.600	25	78.1	26	74.3	0.935
		Butyricicoccus	0.00	0–6.68	0.0	0–0.39	0.219	9	28.1	6	17.1	0.433
		Faecalibacterium	0.0065	0–51.7	0.015	0–3.60	0.420	16	50	20	57.1	0.734
		Oscillospira	0.099	0–8.3	0.051	0–3.48	0.650	25	78.1	27	77.1	0.844
		Ruminococcus	0.0095	0–0.98	0.027	0–0.89	0.350	18	56.3	26	74.3	0.195
		Subdoligranulum	0.024	0–16.6	0.018	0–64.3	0.635	21	65.6	23	65.7	0.803
		Acidaminococcus	0.0	0–0.39	0.0	0–0.69	0.402	10	31.3	7	20	0.438
		Dialister	0.084	0–18.5	0.17	0–10.0	0.728	23	71.9	28	80	0.622
		Megamonas	0.0	0–1.00	0.022	0–2.01	**<0.001** ^†^	9	28.1	25	71.4	**<0.001** ^†^
		Megasphaera	0.31	0–31.9	0.34	0–18.5	0.970	28	87.5	28	80	0.619
		Phascolarctobacterium	0.0	0–0.23	0.0	0–1.76	0.465	7	21.9	10	28.6	0.728
		Veillonella	0.0	0–0.14	0.0	0–0.47	0.804	3	9.4	4	11.4	1.00
	[Mogibacteriaceae]		0.0	0–3.33	0.0	0–0.050	0.653	10	31.3	10	28.6	0.978
		Mogibacterium	0.0	0–1.27	0.0	0–0.93	0.659	6	18.8	8	22.9	0.911
		Anaerococcus	0.0	0–0.027	0.0	0–0.011	0.344	2	6.3	5	14.3	0.431
		Gallicola	0.0	0–0.035	0.0	0–0.026	0.916	4	12.5	5	14.3	1.00
		Parvimonas	0.0	0–0.34	0.0	0–0.061	0.036	13	40.6	6	17.1	0.063
	Erysipelotrichaceae		0.0	0–0.35	0.0	0–0.27	0.427	3	9.4	6	17.1	0.48
		Bulleidia	0.0	0–3.32	0.0	0–4.22	0.805	7	21.9	7	20	0.911
		Catenibacterium	0.0	0–6.28	0.0	0–3.30	0.922	13	40.6	15	42.9	0.950
		Clostridium	0.0	0–0.87	0.0	0–0.32	0.989	14	43.8	16	45.7	0.933
		[Eubacterium]	0.43	0.0054–6.84	0.27	0–8.31	0.660	32	100	32	91.4	0.240
		p-75-a5	0.0	0–0.070	0.0	0–0.043	0.603	4	12.5	3	8.6	0.701
Fusobacteria			0.0	0–10.5	0.0	0–7.87	0.560	15	46.9	17	48.6	0.916
	Fusobacteriaceae		0.0	0–0.19	0.0	0–7.37	0.156	5	15.6	10	28.6	0.329
		Fusobacterium	0.0	0–10.5	0.0	0–1.19	0.954	13	40.6	13	37.1	0.967
Proteobacteria			0.29	0.0088–40.8	0.55	0.019–0–24.7	0.386	32	100	35	100	–
	mitochondria		0.0	0–0.0	0.0	0–0.31	0.256	2	6.3	5	14.3	0.431
		Sutterella	0.0	0–4.5	0.0031	0–2.0	0.261	14	43.8	18	51.4	0.701
	Burkholderiaceae	Burkholderia	0.0	0–0.0	0.0	0–0.071	0.911	7	21.9	8	22.9	0.844
	Comamonadaceae		0.0	0–0.0	0.0	0–0.19	0.982	4	12.5	4	11.4	1.00
		Ralstonia	0.0	0–0.18	0.0	0–0.16	0.318	14	43.8	11	31.4	0.43
		Desulfovibrio	0.0	0–0.0	0.0	0–0.18	0.462	2	6.3	4	11.4	0.675
	Campylobacteraceae	Campylobacter	0.0	0–2.0	0.0032	0–2.37	0.257	12	37.5	19	54.3	0.258
	Helicobacteraceae	Helicobacter	0.0	0–1.1	0.0	0–0.79	0.422	11	34.4	14	40	0.824
		Anaerobiospirillum	0.0	0–1.3	0.0	0–2.51	0.101	6	18.8	12	34.3	0.247
	Enterobacteriaceae		0.051	0–40.7	0.047	0–22.6	0.782	28	87.5	30	85.7	1.00
		Morganella	0.0	0–0.088	0.0	0–0.30	0.562	3	9.4	5	14.3	0.711
	Halomonadaceae	Halomonas	0.0	0–0.0	0.0	0–0.13	0.016	3	9.4	11	31.4	0.055
	Moraxellaceae	Acinetobacter	0.0	0–0.053	0.0	0–0.049	0.306	9	28.1	6	17.1	0.433
		Pseudomonas	0.0022	0–0.23	0.0074	0–0.41	0.625	16	50	20	57.1	0.734
	Vibrionaceae	Vibrio	0.0	0–0.0	0.0	0–0.10	0.028	0	0	5	14.3	0.054
TM7			0.0	0–0.76	0.0	0–0.089	0.135	10	31.3	6	17.1	0.286
	c__TM7-3		0.0	0–0.059	0.0	0–0.008	0.017	7	21.9	1	2.9	0.023
	o__CW040		0.0	0–0.57	0.0	0–0.089	0.244	6	18.8	3	8.6	0.292

Taxa present at a prevalence of < 10% of kittens in both treatment groups are not shown in the table.

† Benjamini-Hochberg corrected p-value < 0.25.

## Discussion

Some unique features of this study are the high-risk target population (shelter kittens), realistic environmental conditions, preventative strategy, naturally-occurring disease outcome measurement, randomized controlled trial design, and use of a host-origin probiotic. Only 2 prior studies describe use of a host-origin probiotic in cats. The first involved administration of *Bifidobacterium pseudocatenulatum* previously isolated from the feces of adult healthy cats to other healthy adult client-owned cats (36). In the second study, *E. faecium* and *Lactobacillus* isolated *via* endoscopic duodenal aspirate from a healthy cheetah, increased body weight and improved fecal quality when given to 8–13 month old cheetah cubs (37).

The present study demonstrates that feline-origin *E. hirae*, administered as a probiotic, is voluntarily consumed by kittens when added to canned cat food, maintained a stable number of colony forming units of bacteria as kept frozen over the course of the study, and had no adverse effects on body weight gain during administration. Based on results of 16S rRNA gene amplicon sequencing, kittens receiving *E. hirae* had a significantly higher relative abundance of *Enterococcus* spp. identified in feces compared to those receiving the placebo. We have previously shown that administration of the same probiotic formulation of *E. hirae* to purpose-bred kittens results in significant increases in the specific identity of *E. hirae* in feces using both qPCR and quantitative culture (25). Inadequate numbers of samples had sufficient DNA remaining after sequencing to perform a specific qPCR assay for *E. hirae* in this study.

The incidence of diarrhea among foster kittens over the course of the study was relatively low. Despite this, there was a significantly lower incidence of diarrhea among kittens receiving the probiotic compared to the placebo which supports a beneficial effect. It is interesting that diarrhea in the placebo group involved a number of litters with multiple affected kittens whereas diarrhea occurred only among singletons in the probiotic group. Individual kittens with diarrhea did not undergo additional diagnostic testing as this was outside the scope of the study. Another limitation of our study is that data on daily fecal consistency of kittens was not obtained. Instead, we chose to conservatively define diarrhea based on a severity prompting examination of the kitten by the shelter veterinary team. This is the precedent means by which the shelter recognizes and treats diarrhea in foster kittens and best represents the subset of kittens at risk for diarrheal morbidity and mortality in the population. An experimental design requiring foster care providers to first witness and then assign a daily fecal score to individual kittens was considered unfeasible and a probable deterrent to participation in the study. Given the lower incidence of diarrhea in kittens receiving the probiotic in this investigation, a targeted study to examine fecal quality of kittens receiving *E. hirae* is of interest.

Our interest in *E. hirae* as a probiotic was based on identification of this species as the predominant mucosa-associated flora in the small intestine of healthy kittens (4). In healthy kittens, *E. hirae* are frequently observed to colonize the epithelial surface (4, 24). Unknown to us at the time of our observation was a fairly robust preexisting attraction to use of *E. hirae* as a probiotic. Numerous isolates of *E. hirae*, ranging in origin from the rumen (38), intestinal tract of ocean (39) and freshwater fish (40) to goats milk (37) have been studied for probiotic effects both *in-vivo* and *in-vitro*. *E. hirae* is bile salt and acid tolerant (41) which promotes its survival through the upper gastrointestinal tract (25) and demonstrate cell wall hydrophobicity which enables it to colonize by interaction with host intestinal epithelial cells (22, 38, 40). A lipoteichoic acid of *E. hirae* has been demonstrated to ameliorate the loss of barrier function caused by the pro-inflammatory cytokine TNF-α on Caco-2 cells by modulating the expression of tight junction regulatory proteins (42). We have previously shown that administration of *E. hirae* to purpose-bred kittens mitigates the increase in intestinal permeability resulting from experimental infection with feline-origin EPEC (25). Other beneficial mechanisms attributed to *E. hirae* include free radical scavenging and lipase activity (17, 40).

We previously observed a decrease in prevalence of mucosa-associated *E. hirae* in the ileum of terminally-ill foster kittens. Loss of *E. hirae* was associated with colonization by virulent genotypes of *E. faecalis* and ability of EPEC to attach to the intestinal epithelium (4). The enterococci, including *E. hirae* (38, 40, 43), produce peptides (i.e., bacteriocins) with a wide range of antibacterial and in some cases antiviral activity (44). Because bacteriocins can promote niche competition with intestinal pathogens, their production is considered to be a favorable attribute of a probiotic (44). For example, when administered to hybrid catfish as a probiotic, *E. hirae* protected against infection by *Aeromonas hydrophila*, enhanced disease protection, and stimulated immunity-related gene expression (45). Similarly, we sought to explore whether administration of feline-origin *E. hirae* probiotic to foster kittens might augment host defense by promoting clearance of existing infectious agents or preventing acquisition of new infectious agents during the course of foster care. To examine this possibility we used a commercial multiplex qPCR-based assay to determine the presence and quantity of intestinal microbes in fecal samples collected prior to onset and upon conclusion of the study. A high prevalence for carriage of several infectious agents was demonstrated prior to onset of the study, which included feline enteric coronavirus, attaching and effacing *E. coli* (presence of *eae*), and panleukopenia virus. A similar prevalence for carriage of these agents in asymptomatic communally housed cats and kittens has been reported by others (46–50). Overall, our study did not provide strong evidence for an impact of *E. hirae* on promoting clearance or preventing acquisition of these selected agents during the course of foster care. The only difference observed between the placebo and *E. hirae* treatment groups was a significant decrease in prevalence of panleukopenia virus DNA among kittens that received the placebo (*p* = 0.002) and a non-significant decrease among kittens that received *E. hirae* (*P* = 0.054). Interpretation of this finding is confounded by concurrent vaccination of kittens with modified-live panleukopenia virus vaccine every 2–3 weeks over the course of the study and the fact that no kittens were clinically unwell at the time of fecal PCR testing. In effort to distinguish between positive PCR results due to modified live virus replication vs. those arising from clinical infection, the reference laboratory applies an a-priori cut-off for positive test results at a Cp value of ≤26. All panleukopenia PCR-positive samples in the study met these criteria. However, recent studies have shown that 21.6% of healthy cats and kittens will shed panleukopenia virus in the positive test result range within 7 days of vaccination (51). Remarkably, the percentage of asymptomatic kittens testing positive for panleukopenia virus DNA in this study is nearly identical at 21.8%. Accordingly it is not unreasonable to conclude that vaccine-origin panleukopenia was largely responsible for PCR-positive results in these healthy kittens. In interpreting the results of the infectious disease testing in this study, it is important to recognize that the presence of microbial DNA does not prove agent viability or measure a potential impact of *E. hirae* on microbial behavior. For example we have previously documented that *E. hirae* can mitigate intestinal injury caused by EPEC infection in kittens while having no measureable impact on EPEC shedding (25).

Based on 16S rRNA gene amplicon sequencing, administration of the *E. hirae* probiotic did not alter the predominant bacterial phyla present in feces which were similar to prior descriptions of the fecal microbiota in cats. These phyla were represented mainly by *Firmicutes, Bacteroidetes, Proteobacteria, Actinobacteria*, and *Fusobacteria* (20, 52–56). The presence of *E. hirae* also did not affect the overall number or diversity of different taxa present in the microbial community which is a desirable attribute of a probiotic. Kittens receiving *E. hirae* had a significant decrease in presence and relative abundance of the genus *Megamonas* (phylum *Firmicutes*, family *Veillonellaceae*). Because *Megamonas* was present at low abundance among the fecal microbiota from kittens, the clinical significance of decreased representation in kittens receiving *E. hirae* is unclear. Arguably the most pertinent location to determine an impact of the *E. hirae* probiotic would be on composition of the small intestinal microbiota where mucosa-associated *E. hirae* dominate the enterococci and from which the probiotic strain was isolated. Obtaining samples from the small intestine of kittens in this study was not feasible using the current study design. In prior reports, dietary supplementation with fermentable prebiotics increased the abundance of *Megamonas* (57–59) while decreased in abundance of *Megamonas* have been described in cats with diarrhea (60). Interestingly, *Megamonas* was increased in feces of cats that were naturally infected with the diarrheal pathogen *Tritrichomonas foetus*, but decreased in feces of cats experimentally infected with the same pathogen (56). These collective observations suggest a vulnerability of *Megamonas* to specific, albeit unidentified changes in the feline intestinal tract. Because the enterococci are lactic acid producers and *Megamonas* utilizes lactate to form propionate (61) we would not have predicted a decrease in *Megamonas* in kittens receiving the *E. hirae* probiotic. In fact, in dogs fed a raw diet, increases in lactate production were positively correlated with abundance of *Megamonas* (62). Ultimately a deeper understanding of any interaction between *E. hirae* and *Megamonas* and the clinical relevance of our observation will require additional study.

In conclusion, a decreased incidence of diarrhea associated with preventative administration of feline-origin *E. hirae* to foster kittens in this study supports a rationale for use of *E. hirae* for disease prevention in this large, young, and vulnerable population.

## Data availability statement

The datasets presented in this study can be found in online repositories. The names of the repository/repositories and accession number(s) can be found below: https://www.ncbi.nlm.nih.gov/, PRJNA776011.

## Ethics statement

The animal study was reviewed and approved by Institutional Animal Care and Use Committee of North Carolina State University (Protocol Number: 18-067-O).

## Author contributions

JG, SJS, JB-B, MA-P, and GD designed the project and secured funding. JG, SHS, SW, EW, and ME performed the experiments. JG, SJS, MA-P, AS, and GD supervised the experiments. JG, SJS, SHS, MA-P, ME, and AS performed bioinformatics analysis. JG, SJS, JB-B, MA-P, ME, AS, and JB interpreted the data. JG, SJS, JB-B, SW, MA-P, ME, AS, and JB wrote sections of the manuscript. All authors have read and approved the final manuscript.

## Funding

This work was supported by grants from the Winn Feline Foundation and PetSmart Charities^®^ (Grant W18-002) and a Research Innovation and Seed Grant from North Carolina State University. The UNC Microbiome Core is supported in part by P30 DK034987 Center for Gastrointestinal Biology and Disease (CGIBD) and the UNC Nutrition Obesity Research Center (NORC P30 DK056350).

## Conflict of interest

Authors ME and AS were employed by IDEXX Laboratories, Inc. Author JB was employed by Vet Med Labor GmbH Division, IDEXX Laboratories, Inc. The remaining authors declare that the research was conducted in the absence of any commercial or financial relationships that could be construed as a potential conflict of interest.

## Publisher's note

All claims expressed in this article are solely those of the authors and do not necessarily represent those of their affiliated organizations, or those of the publisher, the editors and the reviewers. Any product that may be evaluated in this article, or claim that may be made by its manufacturer, is not guaranteed or endorsed by the publisher.
